# Occurrence of chiral organochlorine compounds in the environmental matrices from King George Island and Ardley Island, west Antarctica

**DOI:** 10.1038/srep13913

**Published:** 2015-09-10

**Authors:** Pu Wang, Qinghua Zhang, Yingming Li, Chaofei Zhu, Zhaojing Chen, Shucheng Zheng, Huizhong Sun, Yong Liang, Guibin Jiang

**Affiliations:** 1 State Key Laboratory of Environmental Chemistry and Ecotoxicology, Research Center for Eco-Environmental Sciences, Chinese Academy of Sciences, Beijing 100085, China; 2 Institute of Environment and Health, Jianghan University, Wuhan 430056, China

## Abstract

Chiral organochlorine compounds (OCs) were measured in various environmental matrices (air, soil and vegetation) from west Antarctica using high resolution gas chromatography coupled with high resolution mass spectrometry (HRGC/HRMS). They were generally detected at a global background level compared with the previous studies. α-HCH and PCB-183 was observed in all the matrices except PCB-183 in two soil samples, while PCB-95, -136, -149, -174, -176 and o,p’-DDT were detected in most air but only a few solid matrices. Enantiomeric fractions (EFs) indicated that nonracemic residues of chiral OCs occurred in all the matrices and a wide variation of the EF values was observed in the vegetation. There was significant discrepancy between the EF values of PCB-183 and the racemic values, indicating that stereoselective depletion of PCB-183 was probably associated with the water-air exchange. The EFs values of α-HCH were generally lower than the racemic values but no statistical difference was obtained in all the matrices except lichen, supporting the assumption that water-air exchange may make influence on long-range transport of α-HCH.

Production and use of legacy persistent organic pollutants (POPs), such as polychlorinated biphenyls (PCBs) and organochlorine pesticides (OCPs), have been reduced or eliminated under the Stockholm Convention and some other regional/national initiatives^1^,^2^. These “primary” emissions in environment were effectively curbed, while “secondary” emissions are expected to dominate in the future when these POPs transport and accumulation were controlled by air–surface exchange and the biogeochemical cycle of organic carbon^3^,^4^,^5^. However, it is generally difficult to identify “primary” sources from “secondary” ones by monitoring POPs environmental levels, as well as to demonstrate their transport between the environmental components, i.e., air-surface exchange, even though various mathematical models (e.g., fugacity model) were intensively developed to enhance the understanding of POPs distribution in the environment in the past decades^6^,^7^,^8^,^9^.

Chiral characteristic offers special advantage for tracking transport and fate pathway of POPs in the environment^3^,^4^. In general chiral compounds were commercially manufactured as racemates containing the identical proportions of two enantiomers, of which the enantiomeric ratios (ERs) are constant during physical (e.g., air-water exchange, sorption, volatilization, etc.) and chemical processes (e.g., abiotic reactions). However, biological processes may be enantioselective and yield non-racemic residues, which could be differentiated from fresh source characters on the basis of their enantiomeric composition (i.e., ERs changes). Therefore, it is considered a powerful tool to indicate source characteristics (e.g., “primary” or “secondary”, “new” or “old”, etc.) and gain insights into environmental processes including transport and biotransformation^3^,^4^,^10^,^11^.

Generally, chiral analysis was performed on chromatography and capillary electrophoresis, such as high performance liquid chromatography (HPLC), micellar electrokinetic chromatography (MEKC), capillary electro-chromatography (CEC), supercritical fluid chromatography (SFC), thin layer chromatography (TLC) and gas chromatography (GC)^12^,^13^. Chiral POPs, especially organochlorine compounds (OCs), capillary GC combined with electron capture detector (ECD) or mass spectrometry (MS) was widely employed in the recent years^11^,^14^,^15^,^16^,^17^,^18^,^19^,^20^. Multidimensional and tandem gas chromatographic approaches were also developed and applied for stereoselective analysis of the isomers of some environmental pollutants with increased intensity and selectivity^13^,^20^. Although various attempts were made to completely separate the isomers and stereoisomers in various environmental matrices, it is still a challenge to obtain reliable results at a relatively lower level of the contaminants.

Chiral OCs was previously reported in some remote areas, including Arctic and Antarctica^15^,^16^,^18^,^21^,^22^,^23^,^24^. However, the observations were mostly focused on biota samples where relatively higher concentrations could be measured, and limited data could only be obtained in the environmental matrices in Arctic and nearby areas^22^,^23^. Antarctica is a pristine area with limited human activities. It is considered a final sink for many POPs, and their occurrence has been generally attributed to long-range atmospheric transport (LRAT). Recently, some research works have also proposed additional influence of anthropogenic and inhabitant activities on the elevated levels of POPs in some Antarctic areas^25^,^26^,^27^. This suggests an unexpected and complicated circumstance to evaluate the environmental behavior of POPs in this remote area.

In the present study, high resolution gas chromatography coupled with high resolution mass spectrometry (HRGC/HRMS) was employed to investigate several chiral OCs in the environmental matrices from Fildes Peninsula at King George Island and Ardley Island, west Antarctica. Many international research stations are situated in Fildes Peninsula including Chinese Great Wall Station, while Ardley Island is an important settlement for the penguins and migrating birds. The results of the chiral signatures could therefore contribute to intensively understand the distribution and source of POPs in this pristine area.

## Results

### Chromatographic separation

**Figure 1:**
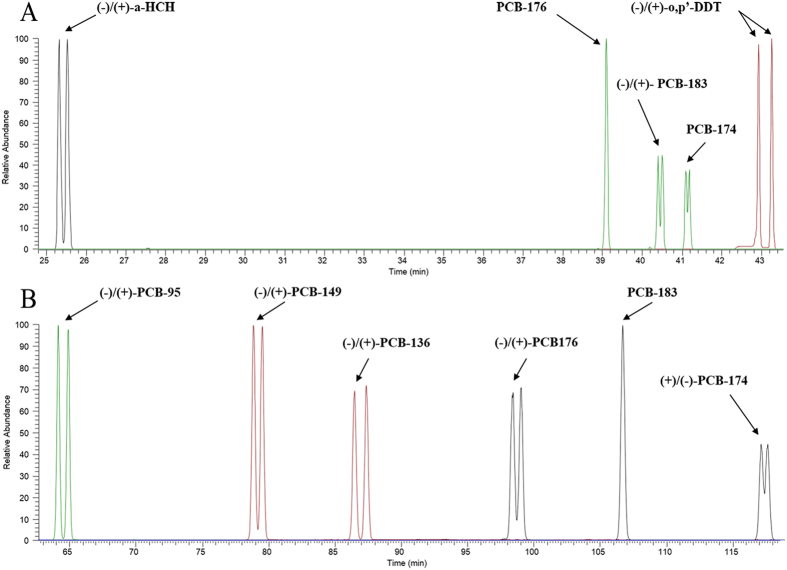
The chromatograms of the chiral resolution of OCs standard solutions on BGB-172(A) and Chiral-Dex (B) columns.

The chromatograms of the chiral resolution of OCs standard solutions were shown in Fig. 1 (A: BGB-172 column and B: Chiral-Dex column). The peak resolution factors (*R*_*s*_) of these chiral compounds were generally high than 1 except for PCB-174 and -83, which could guarantee the chromatographic separation of most target isomers in the present study.

### Overall concentrations

All the chiral OCs could be detected in the air samples except for PCB-176 and *o,p’*-DDT in most cases and PCB-174 in one sample (Table 1). The measured chiral PCBs were at extremely low levels, with a concentration range from not-detected (n.d.) to 0.052 pg m^−3^. Among these congeners, PCB-95 and -136 showed relatively higher levels, confirm to the greater LRAT potential for those lower chlorinated congeners. Besides, α-HCH was measured in the concentration range of 0.12–0.88 pg m^−3^, which is relatively higher than the levels of chiral PCBs in air. In the other matrices, PCB-183 was measured in the most samples with concentration ranges from n.d. to 4.71 pg g^−1^ dry weight (dw) in soil and sediment, and 0.77 to 7.05 pg g^−1^ dw in vegetation. PCB-174 was also found in the most vegetation (n.d. −4.57 pg g^−1^ dw) and half of the soil samples (n.d. −9.95 pg g^−1^ dw). While PCB-95 and −136 accounted for a relatively lower detection rate (about 40%) and concentration level (n.d. −2.64 pg g^−1^ dw), PCB-149 and -176 were even not detected in any of the samples. In contrast, α-HCH concentrations were relatively higher with a range of 4.36–54.7 pg g^-1^ dw in soil and sediment, and 16.4–69.2 pg g^−1^ dw in the vegetation samples. Comparable to the observation in the air samples, *o,p’*-DDT was not detected in any of the solid samples.

### Chiral signature

**Figure 2:**
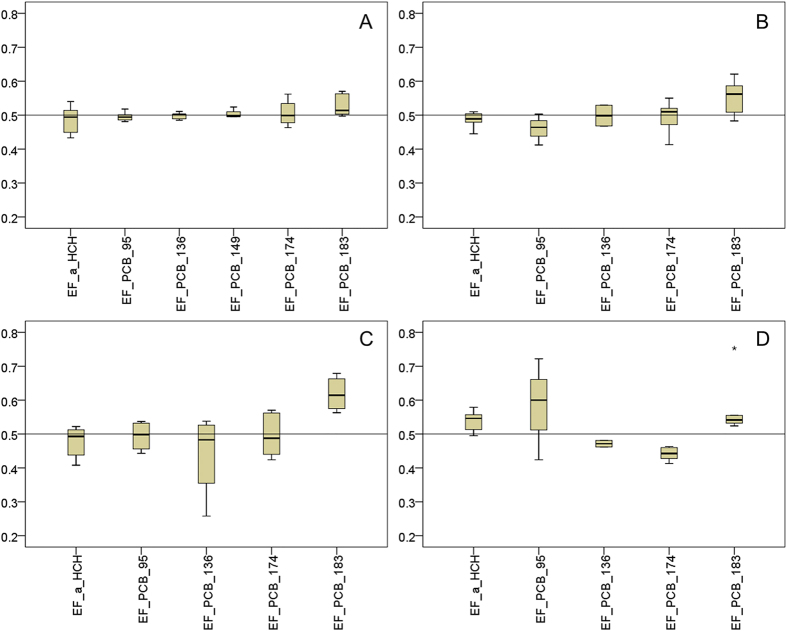
Enantiomeric fractions (EFs) of detected OCs in air (A), soil (B), moss (C) and lichen (D). The line represents the theoretical EF of racemic mixtures (0.500). Extreme value (marked with a black star) was beyond the triplication of the difference between the 25th and 75th percentile.

Enantiomeric fraction (EF)^28^ was calculated based on the peak areas of (+) and (−) stereoisomers, which was illustrated in Fig. 2. For chiral PCBs, PCB-183 showed generally higher EF values (mean 0.529 in air, 0.552 in soil, 0.575 in lichen and 0.611 in moss) compared to the other chiral compounds. Significant difference was obtained between the EF values of chiral PCB-183 and those in the racemic standard (*p* < 0.05), suggesting a nonracemic residue of PCB-183 in all the matrices and occurrence of preferential depletion of (-) PCB-183. Moreover, an average close to 0.500 was obtained for PCB-95, -136, -149 and -174 in the air samples (Fig. 2A), while most EF values fell outside the 95% confidence intervals of those of racemic standard, indicating that nonracemic residue of chiral PCBs could still be observed in the air. As well, the EFs of chiral PCBs in the soil and vegetation generally showed a wide variation, especially in the lichen, the higher-chlorinated chiral compounds (e.g., PCB-136, -174 and -183) had significantly nonracemic signatures (*p* < 0.04 when compared to EFs of racemic standard), suggesting stereoselective depletion of chiral PCBs in the Antarctic environment.

EFs of α-HCH were 0.433–0.540 (mean 0.486) in air, 0.445–0.510 (mean 0.488) in soil, 0.499 in the sediment, 0.495–0.579 (mean 0.539) in lichen and 0.408–0.532 (mean 0.477) in moss, respectively. There were no significant differences between these values and the EFs of racemic standard (*p* > 0.05) except for lichen, of which the EF ratios were significantly higher (*p* = 0.004). This indicated nonracemic residue of α-HCH in the lichen, comparable to the observation of chiral PCBs. Moreover, significant discrepancy was observed between the EFs in lichen and moss (*p* = 0.011), supporting the assumption of interspecific difference in enantioselective transformation of chiral α-HCH.

## Discussion

The concentration levels of chiral PCBs were in agreement with those of dioxin-like PCBs (dl-PCBs) according to our previous report^29^,^30^ and the study by Choi *et al.*^25^ in the West Antarctica. There was no report involving chiral PCBs measurement in Antarctica as far as we know. While our recent study^22^ in Svalbard, Arctic, showed that chiral PCBs (PCB-95, -136, -149, -174 and -176) could be detected in all the analyzed matrices (soil, reindeer dung and six plant species) except for PCB-174 in reindeer dung and PCB-176 in one of the plant species (*D. octopetala*). Comparable to the low level of chiral PCBs in Arctic, the present results revealed a global background level of these contaminants in the west Antarctica. For α-HCH, the levels in air was consistent with the observations in Ross Sea^31^ and west Antarctic Peninsula^32^, but lower by at least one order of magnitude than those in arctic areas in recent years^33^,^34^. In the other solid matrices, they were also generally in agreement with many previous studies in Antarctica. For instances, Cipro *et al.*^35^ found that HCHs ranged from <MDL (method detection limit) to 1.20 ng g^−1^ dw in vegetation from King George Island; Borghini *et al.*^36^ observed that α-HCH concentrations were <0.01–0.026 ng g^−1^ dw in soils and 0.43–4 ng g^−1^ dw in mosses from Victoria land, Antarctica. Whereas, relatively higher levels were also reported in some research works, e.g., Zhang *et al.*^37^ obtained that α-HCH concentrations were in the range of 0.17–0.65 ng g^−1^ on habitats of seabirds and seal in Fildes Peninsula and Ardley Island; Klánová *et al.*^38^ reported the concentrations of HCHs isomers ranged between 0.49 and 1.34 ng g^−1^ in soil samples from James Ross Island, Antarctica. These disparities could be due to the different sampling sites and sampling period.

α-HCH is one of the most extensively investigated chiral isomer in the environment. Preferential degradation of (+) α-HCH was observed in most cases, by virtue of enantiomeric signatures, especially in the Arctic region^4^,^13^. Similar phenomenon was observed in the present study with relatively lower mean EFs in all the matrices except lichen. Although Kurt-Karakus *et al.*^39^ found an EF of 0.508 in the Antarctica soil, it still fell inside the range of our result. Enantiomeric depletion of chiral α-HCH in Antarctic biota was also observed by Corsolini *et al.*^21^. The average EF values were 0.44 ± 0.01, 0.49 ± 0.01 and 0.58 ± 0.04 in krill, emerald rockcod and Adélie penguin eggs, respectively, which suggested preferential depletion of (+) α-HCH in the lower trophic levels and enantioselective biotransformation in the food web. In the present study, the enantioselective biotransformation of α-HCH could also be observed in the dropping amended soil samples, while the EF values were generally lower than 0.500, indicating preferential biotransformation of (+) α-HCH in animal metabolism and the influence of animal activities on the POPs distribution.

Some previous studies observed correlations between the EF values and concentrations of chiral compounds, e.g., Asher *et al.*^40^ found the EFs of PCB-95 in total suspended matters of Upper Hudson River as a function of total PCB concentrations; Huang *et al.*^17^ observed a strongly relationship between *o,p′*-DDT concentrations and its EFs in the equatorial Indian Ocean and near racemic compositions were found in the sites with high concentrations. In the present study, we examined the relationship between the EFs and concentrations (as well as the deviation from racemic (DFR) which was defined as |0.500-EF|^41^) of α-HCH and PCB-183, both of which were evidently detected in the samples, while we did not found any correlations (Figures S1, S2 in Supplementary information). This might be due to the low level of concentrations and wide variation of EFs in the samples.

In general, primary source yields a similar enantiomeric signature of chiral compounds to the racemic mixture, while chiral OCs emission from secondary sources associates with microbial transformation in soil and water, inducing variation of the EFs in atmosphere through water-air or soil-air exchange^4^. In this study, chiral OCs exhibited a comparable profile of EF ratios in the air samples to that in the soil (*p* > 0.05), and most of which were close to the racemic values (excluding PCB-183). Considering that the sampling area is much smaller compared to the surrounding ocean and most of the area is perennially covered by snow, soil-air exchange could not obviously affect the distribution of POPs in the west Antarctic ambient air. Meanwhile, nonracemic residue of PCB-183 was observed in all the matrices with generally higher EF values (*p* < 0.05), indicating that stereoselective depletion of PCB-183 was probably associated with the water-air exchange.

Many previous works have shown an enantioselective degradation of α-HCH in polar seas. For example, Jantunen *et al.*^42^ investigated EF ratios of α-HCH in seawater collected during the South African National Antarctic Expedition, the values ranged from 0.477 to 0.515 and evidenced a significant regression of EF versus latitude (R^2^ = 0.28, *p* ≤ 0.005). Extensive studies in the Arctic Ocean have demonstrated a preferential degradation of (+) α-HCH in seawater^28^,^43^. In the present study, the results showed generally lower EF values of α-HCH than the racemic values in all the matrices except lichen, indicating enantioselective degradation of (+) α-HCH. This is comparable to the observation of chiral PCB-183, shedding light on somewhat influence of water-air exchange on the LRAT of α-HCH to this remote area.

In conclusion, this study revealed occurrence of the typical chiral OCs in various environmental matrices from west Antarctica. The results indicated a global background level of these compounds in the air, soil and vegetation samples. The enantiomer signatures indicated nonracemic residues of chiral OCs in the matrices and limited influence of soil-air exchange on the distribution of chiral OCs in the west Antarctic environment. Nevertheless, water-air exchange and animal activity may affect their enantiomer composition during their long-range transport and redistribution in the remote area. Since this is the first attempt at chiral PCBs measurement in the Antarctic environment to our best knowledge, further investigation is worth carrying out to assess the source implication of chiral signatures of OCs in this remote area.

## Methods

### Sample collection

A total of thirty environmental samples were collected around Fildes Peninsula at King George Island where many international research stations are situated including Chinese Great Wall Station, and Ardley Island which is an important settlement for the penguins and migrating birds. All the samples, including five airs collected by PUF passive air sampling, ten soils (seven natural soils and three dropping-amended soils), one sediment, six lichens and eight mosses, were stored under −20 ^o^C after being transported back to the laboratory. Sampling details and sample characteristics were introduced in our previous works^29^,^30^.

### Sample analysis

The sample extraction and cleanup procedures were followed our previous methods with minor modification^29^,^30^,^44^. Instrumental analysis was performed on DFS system (Thermo Fisher, USA) at a resolution ≥8,000. Chiral analysis was carried out on BGB-172 (30 m×0.25 mm i.d. ×0.25 μm thickness, BGB Analytik AG, Switzerland) and Chirasil-Dex (30 m×0.25 mm i.d. ×0.25 μm thickness, Varian, Walnut Creek, CA) columns. The details were shown in Supplementary information.

### Statistical analyses

SPSS 20.0 was employed for statistical analyses of the results. The significant difference was evaluated between the EF values in the samples and those of racemic standards using independent samples t-test, as well as the EFs between the matrices. Statistical significance was determined at both the confidence levels of 0.01 and 0.05.

## Additional Information

**How to cite this article**: Wang, P. *et al.* Occurrence of chiral organochlorine compounds in the environmental matrices from King George Island and Ardley Island, west Antarctica. *Sci. Rep.* **5**, 13913; doi: 10.1038/srep13913 (2015).

## Supplementary Material

Supplementary Information

## Figures and Tables

**Table 1:** The mean concentrations (range) of chiral OCs in the environmental samples from west Antarctica.

	Air (n = 5) (pg m^−3^)	Soil(n = 10) (pg g^−1^ dw)	Sediment(n = 1) (pg g^−1^ dw)	Moss(n = 8) (pg g^−1^ dw)	Lichen(n = 6) (pg g^−1^ dw)
PCB-95	0.018(0.004–0.042)	0.26(n.d.* −1.49)	1.08	0.55(n.d. −1.45)	0.14(n.d. −0.37)
PCB-136	0.034(0.014–0.052)	0.13(n.d. −0.94)	1.19	0.63(n.d. −2.64)	0.09(n.d. −0.27)
PCB-149	0.010(0.002–0.016)	n.d.	n.d.	n.d.	n.d.
PCB-174	0.003(n.d. −0.005)	1.47(n.d. −9.95)	n.d.	1.65(n.d. −4.57)	1.60(0.63–4.23)
PCB-176	0.001(n.d. −0.004)	n.d.	n.d.	n.d.	n.d.
PCB-183	0.012(0.004–0.018)	1.75(n.d. −4.71)	3.12	3.79(1.54–7.05)	1.67(0.77–3.53)
α-HCH	0.32(0.12–0.88)	13.7(4.36–54.7)	54.3	29.1(16.4–51.2)	43.6(23.4–69.2)
*o,p’*-DDT	0.008(n.d. −0.019)	n.d.	n.d.	n.d.	n.d.

^*^n.d., not detected.
