# Corrigendum: Occurrence of chiral organochlorine compounds in the environmental matrices from King George Island and Ardley Island, west Antarctica

**DOI:** 10.1038/srep16549

**Published:** 2015-12-18

**Authors:** Pu Wang, Qinghua Zhang, Yingming Li, Chaofei Zhu, Zhaojing Chen, Shucheng Zheng, Huizhong Sun, Yong Liang, Guibin Jiang

Scientific Reports 5: Article number: 13913; 10.1038/srep13913 published online: 09102015; updated: 12182015.

In the original version of this Article, Affiliation 2 was omitted for Qinghua Zhang.

In addition, there were typographical errors.

In the Results section under subheading ‘Chromatographic separation’

“The peak resolution factors (*R*_*s*_) of these chiral compounds were generally high than 1 except for PCB-174 and -83”

now reads:

“The peak resolution factors (*R*_*s*_) of these chiral compounds were generally higher than 1 except for PCB-174 and -183”

In the Methods Section under subheading ‘Sample Analysis’

“Chiral analysis was carried out on BGB-172 (30  m×0.25  mm i.d. ×0.25 μm thickness, BGB Analytik AG, Switzerland) and Chirasil-Dex (30  m×0.25  mm i.d. ×0.25 μm thickness, Varian, Walnut Creek, CA) columns.”

now reads:

“Chiral analysis was carried out on BGB-172 (30 m×0.25 mm i.d. ×0.25 μm thickness, BGB Analytik AG, Switzerland) and Chirasil-Dex (25 m×0.25 mm i.d. × 0.25 μm thickness, Varian, Walnut Creek, CA) columns.”

These errors have now been corrected in the PDF and HTML versions of the Article.

And lastly, in the Supplementary Information file originally published with this Article, there was a typographical error in the Experimental Data section under subheading ‘Chiral analysis’.

“While the other chiral OCs (PCB-95, -136, -149, -174 and -176) were separated on Chirasil-Dex (30 m×0.25 mm i.d. ×0.25 μm thickness, Varian, Walnut Creek, CA)”

now reads:

“While the other chiral OCs (PCB-95, -136, -149, -174 and -176) were separated on Chirasil-Dex (25 m×0.25 mm i.d. ×0.25 μm thickness, Varian, Walnut Creek, CA)”.

This error has been corrected in the Supplementary Information that now accompanies the Article.

