# Supplementary material for: Occurrence of chiral organochlorine compounds in the environmental matrices from King George Island and Ardley Island, west Antarctica
Source: Sci Rep. 2015 Sep 10;5:13913. doi: 10.1038/srep13913 (PMC4564805; doi:10.1038/srep13913)

Supplementary Information

Occurrence of chiral organochlorine compounds in the environmental matrices from King George Island and Ardley Island, west Antarctica

Pu Wang[1], Qinghua Zhang[1],* Yingming Li[1], Chaofei Zhu[1], Zhaojing Chen[1], Shucheng Zheng[1], Huizhong Sun[1], Yong Liang[2] & Guibin Jiang[1]

[1]State Key Laboratory of Environmental Chemistry and Ecotoxicology, Research Center for Eco-Environmental Sciences, Chinese Academy of Sciences, Beijing 100085, China

[2]Institute of Environment and Health, Jianghan University, Wuhan 430056, China

*Corresponding author: Qinghua Zhang

State Key Laboratory of Environmental Chemistry and Ecotoxicology,

Research Center for Eco-Environmental Sciences, Chinese Academy of Sciences,

Beijing 100085, China.

Fax/Tel: 86-10-62849818

E-mail: qhzhang@rcees.ac.cn

Figure Caption:

Figure S1. The scatter plot of α-HCH concentrations versus its EF values.

Figure S2. The scatter plot of PCB-183 concentrations versus its EF values.

Experimental Data

Materials Pesticide-grade reagents dichloromethane (DCM) and *n*-hexane, HPLC-grade acetonitrile were purchased from J.T. Baker (Phillipsburg, NJ, USA). Nonane was from Sigma-Aldrich (St. Louis, USA). Silica gel 60 (0.063-0.100 mm particle diameter) was obtained from Merck (Darmstadt, Germany). C_{18} SPE column (LC-18 SPE，1 g/6mL) was supplied by Supelco (Bellefonte, PA, USA). Anhydrous sodium sulfate and concentrated sulfuric acid were of guaranteed grade from domestic market (purity >99.8%). Prior to use, silica gel and anhydrous sodium sulfate were baked at 550 ^{o}C for 12 h and 660 ^{o}C for 6 h, respectively. The stock solutions of chiral isomers including PCB-95, -136, -149, -174, -176, -183, α-HCH and *o,p'*-DDT were supplied by AccuStandard Inc. (New Haven, CT, USA). The ^{13}C-labeled internal standards (EPA 68B-LCS) and injection standards (EPA 68B-IS) were purchased from Wellington Laboratories (Guelph, Ontario, Canada).

Sample extraction and cleanup The analytical procedure was followed our previous methods with minor modification[1-3]. Air samples and the other freeze-dried environmental samples were spiked with EPA 68B-LCS (including ^{13}C-PCB-15, -54, -155, -167, -189 and -202) prior to extraction. Approximately 10 g soil (or sediment) sample was weighed and extracted with DCM: *n*-hexane (1:1, v:v) on Accelerated Solvent Extraction (ASE300, Dionex, USA), while 3 g vegetation sample and a whole PUF-disk were extracted for chiral OCs measurement. The extraction condition was as follows: temperatures were 150 ^{o}C for soil and 100 ^{o}C for vegetation and air samples; pressure was 1500 psi (10.3 MPa); heating time was 7 min and followed by 8 min in static state; extraction cycle was set as 3 times. The extract was concentrated and loaded to a multilayer silica gel column (from bottom up: 1 g silica gel, 4 g 22% (w/w) acidified silica gel, 1 g silica gel, 8 g 44% (w/w) acidified silica gel, 2 g silica gel and 2 cm anhydrous sodium sulfate), and then followed by a C_{18} SPE column. The multilayer column was pre-washed using 80 mL *n*-hexane and the targets were eluted with 100 mL *n*-hexane. While C_{18} SPE column was rinsed and eluted with 6 mL and 12 mL acetonitrile, respectively. The eluate was finally evaporated to dryness and solvent-exchanged with *n*-hexane. It was finally concentrated under a stream of

nitrogen flow into 20 μL nonane in a GC vial. Prior to instrumental analysis, the solution was spiked with EPA 68B-IS (including ^{13}C-PCB-52, -101, -138 and -194) for recovery calculation.

Chiral analysis The instrumental analysis was performed on DFS system (Thermo Fisher, USA) in positive electron ionization (EI+). The HRMS was operated in the multiple ion detection (MID) mode at a resolution ≥8,000. The electron emission energy was set as 45 eV, emission current was 0.9 mA and acceleration voltage was 4800 *V*. Perfluorotributylamine (FC43) was used for the calibration of mass spectrometers and two most abundant ions of the molecular ion cluster for target isomer were monitored. The ion source temperature was optimized over a temperature interval of 220-270 ^{o}C, 240 ^{o}C and 250 ^{o}C was finally selected for chiral OCs analysis on BGB-172 and Chirasil-Dex columns, respectively.

BGB-172 (20% tertbutyldimethylsilylated b-cyclodextrin in OV-1701, 30 m×0.25 mm i.d. ×0.25 μm thickness, BGB Analytik AG, Switzerland) was mainly employed for enantiomers separation of α-HCH, *o,p*'-DDT and PCB-183. Helium was the carrier gas with a constant flow of 1.5 mL min^{-1}. The GC temperature program was: initial temperature 100 ^{o}C, then 4 ^{o}C min^{-1} to 180 ^{o}C, 2 ^{o}C min^{-1} to 200 ^{o}C, 4 ^{o}C min^{-1} to 245 ^{o}C and finally hold for 9 min. The temperature of injection port and transfer line was set as 240 ^{o}C. While the other chiral OCs (PCB-95, -136, -149, -174 and -176) were separated on Chirasil-Dex (30 m×0.25 mm i.d. ×0.25 μm thickness, Varian, Walnut Creek, CA). The carrier gas (helium) was at a constant flow of 1.0 mL min^{-1}. The GC temperature programming condition was as follows: the initial temperature was held at 60 ^{o}C for 1 min and increased to 150 ^{o}C at 5 ^{o}C min^{-1}, then ramped to 192 ^{o}C at 0.4 ^{o}C min^{-1}, and finally reached to 250 ^{o}C at 15 ^{o}C min^{-1}. The temperature for the GC injection port and transfer line was selected as 260 ^{o}C.

Enantiomeric fractions (EFs)[4] were calculated as follows:

$$EF = \frac{(+)}{(+)+(-)} \qquad (1)$$

Peak areas of (+) and (-) stereoisomers were used of which stereoisomer elution order was identified (e.g., α-HCH, *o,p'*-DDT and PCB-183 on BGB-172 column[5-8], PCB-95, -136, -149, -174 and -176 on Chirasil-Dex column[6,9-10]).

Quality assurance/quality control HRGC/HRMS were employed for determination of chiral OCs. Quantification was performed based on sum of the peak areas of the two monitored ions. The ratios between the ion pairs were defined within ±15% of the theoretical abundance ratio. The peak resolutions of these chiral compounds were generally high than 1 except for PCB-174 and -183 (Figure 1). Calibration curve was established over the concentration interval of 0.5-500 ng mL^{-1} and the relative standard deviation (RSD) for the average response factor (RF) of each compound was less than 15%. All the samples were spiked with ^{13}C-labeled internal standards 68B-LCS (EPA including ^{13}C-PCB-15, -54, -155, -167, -189 and -202) for quantification and 68B-IS (including ^{13}C-PCB-52, -101, -138 and -194) for recovery calculation. The recoveries of 68B-LCS were 62.2±20.3%, which could guarantee the analytical quality according to EPA method 1668A and method 1699. A sample of XAD resins was used as travel blank to monitor the contamination, and three laboratory blanks were processed paralleled to the samples analysis for quality control. There were no chiral compounds detected in all the blanks. Limits of detection (LOD) was defined as signal-to-noise ratio (S/N) = 3:1. The LODs for chiral OCs (except *o,p'*-DDT) were in the range of 0.0004-0.0077 pg m^{-3} in air, 0.03-1.19 pg.g^{-1} in vegetation, 0.01-1.07 pg g^{-1} in soil and sediment, respectively. While LODs for *o,p'*-DDT were generally higher than 0.0072 pg m^{-3} in air and 1.09 pg g^{-1} in the other matrices. The LOD values were one or two orders of magnitudes lower than those in many previous studies in the polar areas[11-15].

Racemic standards were repetitive analyzed to determine the reproducibility of EF measurements (n=8). Average EF values were 0.501±0.001, 0.497±0.003, 0.501±0.003, 0.498±0.002, 0.499±0.002, 0.503±0.006, 0.508±0.002, 0.504±0.002 for α-HCH, *o,p'*-DDT, PCB-95, -136, -149, -174, -176 and -183, respectively. The 95% confidence intervals of their EF values were 0.499-0.503, 0.489-0.505, 0.495-0.508, 0.492-0.504, 0.494-0.504, 0.489-0.518, 0.502-0.514 and 0.498-0.508, respectively. If the EF for an individual sample fell inside these ranges for the standards, they were interpreted as not significantly different from racemic[16]. A commercial formulation (Aroclor 1260 contains more high-chlorinated PCB congeners) was analyzed to check

if there was any interference existed around the target chiral compounds. Wong et al.[17] indicated the target compounds could be successfully separated on one chiral capillary column except PCB-95 which was interfered with a coeluting pentachlorobiphenyl on Chirasil-Dex column. In our study, we did not observed evident interference around the chromatographic peak of PCB-95, as well as the other target chiral compounds, which could ensure that there was no evident coelution of chiral PCBs with other congeners.

Reference

1. Li, Y. M. *et al.* Study of PCBs and PBDEs in King George Island, Antarctica, using PUF passive air sampling. *Atmos. Environ.* **51**, 140-145 (2012).
2. Wang, P. *et al.* PCBs and PBDEs in environmental samples from King George Island and Ardley Island, Antarctica. *RSC Adv.* **2**, 1350-1355 (2012).
3. Chen, Z. J. *et al.* Determination of organochlorine pesticides in Antarctic samples using high resolution gas chromatography/high resolution mass spectrometry (HRGC/HRMS). *Environ. Chem.* (in Chinese) **33**, 1655-1661 (2014).
4. Harner, T., Wiberg, K. & Norstrom, R. Enantiomer fractions are preferred to enantiomer ratios for describing chiral signatures in environmental analysis. *Environ. Sci. Technol*. **34**, 218-220 (2000).
5. Falconer, R. L., Bidleman, T. F. & Szeto, S. Y. Chiral pesticides in soils of the Fraser Valley, British Columbia. *J. Agr. Food Chem.* **45**, 1946-1951 (1997).
6. Wong, C. S. Enantiomer fractions of chiral organochlorine pesticides and polychlorinated biphenyls in standard and certified reference materials. *Chemosphere* **49**, 1339-1347 (2002).
7. Toda, M. *et al.* Absolute configuration of atropisomeric polychlorinated biphenyl 183 enantiomerically enriched in human samples. *J. Phys. Chem. A*. **116**, 9340-9346 (2012).
8. Kania-Korwel, I. & Lehmler, H. J. Assigning atropisomer elution orders using atropisomerically enriched polychlorinated biphenyl fractions generated by microsomal metabolism. *J. Chromatogr. A*. **1278**, 133-144 (2013).
9. Dai, S. *et al.* Enantioselective accumulation of chiral polychlorinated biphenyls in lotus plant (Nelumbonucifera spp.). *J. Hazard Mater.* **280**, 612-618 (2014).
10. Wong, C. S., Garrison, A. W. & Foreman, W. T. Enantiomeric composition of chiral polychlorinated biphenyl atropisomers in aquatic bed sediment. *Environ. Sci. Technol*. **35**, 33-39 (2001).
11. Hoekstra, P. F., O'Hara, T. M., Karlsson, H., Solomon, K. R. & Muir, D. C. Enantiomer-specific biomagnification of alpha-hexachlorocyclohexane and selected chiral chlordane-related compounds within an Arctic marine food web. *Environ. Toxicol. Chem.* **22**, 2482-2491 (2003).
12. Hoekstra, P. F. *et al.* Profile of persistent chlorinated contaminants, including selected chiral compounds, in wolverine (Gulo gulo) livers from the Canadian Arctic. *Chemosphere* **53**, 551-560 (2003).
13. Negoita, T. G., Covaci, A., Gheorghe, A. & Schepens, P. Distribution of polychlorinated biphenyls (PCBs) and organochlorine pesticides in soils from the East Antarctic coast. *J. Environ. Monitor.* **5**, 281-286 (2003).

14. Borghini, F., Grimalt, J. O., Sanchez-Hernandez, J. C. & Bargagli, R. Organochlorine pollutants in soils and mosses from Victoria Land. *Chemosphere* **58**, 271-278 (2005).
15. Bengtson Nash, S. M., Poulsen, A. H., Kawaguchi, S., Vetter, W. & Schlabach, M. Persistent organohalogen contaminant burdens in Antarctic krill (Euphausia superba) from the eastern Antarctic sector: A baseline study. *Sci. Total Environ.* **407**, 304-314 (2008).
16. Genualdi, S. A. *et al.* Enantiomeric signatures of organochlorine pesticides in Asian, trans-Pacific, and Western U.S. air masses. *Environ. Sci. Technol.* **43**, 2806-2811 (2009).
17. Wong, C. S. & Garrison, A. W. Enantiomer separation of polychlorinated biphenyl atropisomers and polychlorinated biphenyl retention behavior on modified cyclodextrin capillary gas chromatography columns. *J. Chromatogr. A.* **866**, 213-220 (2000).

Figure S1. The scatter plot of α-HCH concentrations versus its EF values.

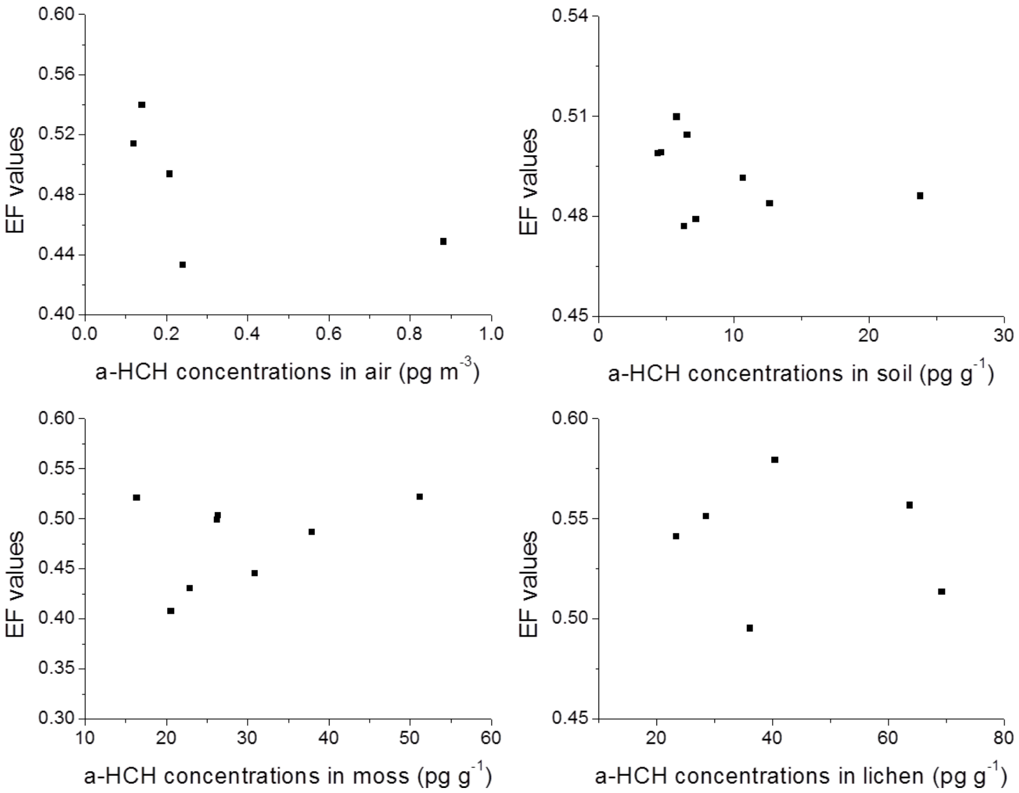

Figure S2. The scatter plot of PCB-183 concentrations versus its EF values.

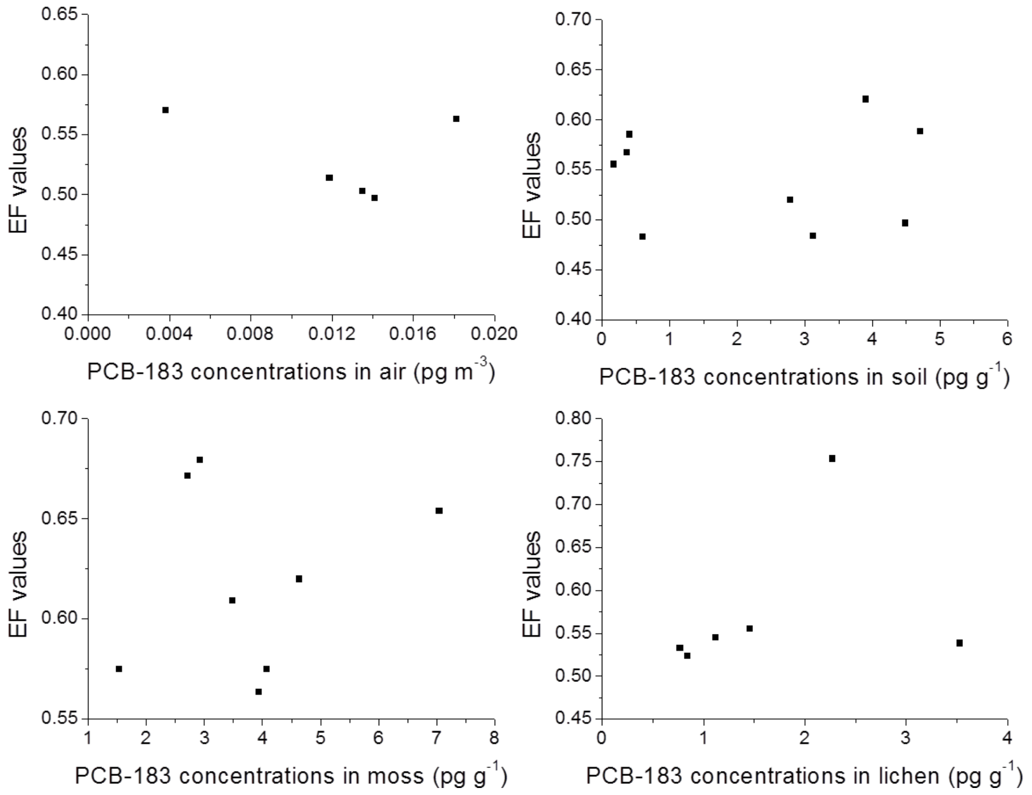

Supplement: Supplementary Information [file srep13913-s1.pdf]
